# The Nif3-Family Protein YqfO03 from *Pseudomonas syringae* MB03 Has Multiple Nematicidal Activities against *Caenorhabditis elegans* and *Meloidogyne incognita*

**DOI:** 10.3390/ijms19123915

**Published:** 2018-12-06

**Authors:** Abdul Manan, Zahoor Ahmad Bazai, Jin Fan, Huafu Yu, Lin Li

**Affiliations:** 1 State Key Laboratory of Agricultural Microbiology, College of Life Science and Technology, Huazhong Agricultural University, Wuhan 430070, China; manan_haqyar@yahoo.com (A.M.); fanjin3874@163.com (J.F.); yuhuafu20093919@163.com (H.Y.); 2 Center for Advance Studies in Vaccinology and Biotechnology, University of Baluchistan, Quetta 87300, Pakistan; 3 Department of Botany, University of Baluchistan, Quetta 87300, Pakistan; z_Bazai@yahoo.com

**Keywords:** *Pseudomonas syringae*, Nif3-family protein, virulence factor, nematicidal activity, *Caenorhabditis elegans*, *Meloidogyne incognita*

## Abstract

The nematicidal activity of the common plant-pathogenic bacterium *Pseudomonas syringae* against certain nematodes has been recently identified, but little is known about its virulence factors. In the current study, predictive analysis of nematode-virulent factors in the genome of a *P. syringae* wild-type strain MB03 revealed a variety of factors with the potential to be pathogenic against nematodes. One of these virulence factors that was predicted with a high score, namely, YqfO03, was a protein with structural domains that are similar to the Nif3 superfamily. This protein was expressed and purified in *Escherichia coli*, and was investigated for nematicidal properties against the model nematode *Caenorhabditis elegans* and an agriculturally important pest *Meloidogyne incognita*. Our results showed that YqfO03 exhibits lethal activity toward *C. elegans* and *M. incognita* worms, and it also caused detrimental effects on the growth, brood size, and motility of *C. elegans* worms. However, *C. elegans* worms were able to defend themselves against YqfO03 via a physical defense response by avoiding contact with the protein. Discovery of the diverse nematicidal activities of YqfO03 provides new knowledge on the biological function of a bacterial Nif3-family protein and insight into the potential of this protein as a specific means of controlling agricultural nematode pests.

## 1. Introduction

A variety of soil-borne nematodes can infect the plants and inflict serious damage to plant hosts. Various pathogenic nematodes, including plant parasitic nematodes (PPNs), species in the genera of *Meloidogyne*, *Anguina*, *Heterodera*, *Bursaphelenchus*, *Ditylenchus,* and *Pratylenchus* dominate global damages in agricultural and forest production that results in huge economic losses each year [1,2]. One of the soil-transmitted PPNs, the root-knot nematode *M. incognita*, is able to infect the roots of almost all cultivated plants [2,3,4]. Various strategies have been used to control such harmful nematodes. Conventional approaches to controlling nematodes use different chemical nematicides. However, increasing concern about human and environmental safety has restricted the use of nematicides [5]. Current efforts to protect crops from nematode infestation focus on environmentally safe products [6]. In this respect, many nematicidal bacteria have shown usefulness in controlling the detrimental effects of certain nematode pathogens, including PPNs [7]. These bacteria include *Pasteuria penetrans* [8], *Pseudomonas aeruginosa* [9,10], *Bacillus thuringiensis* [11], *Bacillus firmus* [12], *Paecilomyces lilacinus* [13], *Arthrobotrys oligospora* [14], and *Burkholderia cepacia* [15], among the others. These microorganisms affect nematodes via different mechanisms, such as parasitism and antibiotic production and by producing toxins or enzymes that impede plant-host recognition by the nematode [7]. Such mechanisms raise the possibility that nematicidal bacteria could be used to prevent the plant damage that is caused by PPNs [16,17].

The Gram-negative *Pseudomonas* is a highly diverse bacterial genus consisting of many pathogenic species, including the human opportunistic pathogen *Pseudomonas aeruginosa* and the plant pathogen *Pseudomonas syringae* [18,19]. Previous studies to elucidate the virulence mechanisms of *P. aeruginosa* against the nematode *Caenorhabditis elegans* have shown that a variety of proteins are involved in the virulence of *P. aeruginosa* against this worm [20,21,22]. *P. syringae* is generally recognized as a conventional plant pathogen; however, as more genomic sequences of *P. syringae* are being released, it is becoming clear that many of the virulence genes that were found in *P. syringae* are identical to those found in the *P. aeruginosa* genome [23,24,25]. Therefore, *P. syringae* has nematicidal potential against *C. elegans* in view of nematode-virulent genes in its genome. For the nematicidal activity analysis of naturally occurring nematicidal bacteria or genetically expressed virulence factors, the free living nematode, *C. elegans*, has been used extensively as a target nematode due to the well-known genetic background of *C. elegans*, facile cultivation processes, and short lifecycle for facilitating bioassays [26]. In fact, we demonstrated the pathogenicity of a *P. syringae* wild-type strain MB03 against *C. elegans* in a recent study [25]. However, the factors that are responsible for this pathogenicity are still unknown. The present study was aimed to assay the nematicidal activity of an NGG1p interacting factor 3 (Nif3, in brief) like protein, YqfO03, from *P. syringae* MB03 against *C. elegans* and *M. incognita*. The Nif3 family proteins have been found in all three domains of life, but the precise function of this protein remains uncertain in bacterial cells [27]. A recent study has revealed that one Nif3-related protein from *Bacillus subtilis* was highly likely to be responsible for the inhibitory effect on soil-borne plant pathogen *Fusarium oxysporum* in the early phase of recognition in addition to the known cell wall-degrading enzymes [28]. This provides a hint that the Nif3 protein could be a prohibitive factor restricting the infestation of certain pathogens. Our results showed that the Nif3-like protein from *P. syringae* had toxicity against *C. elegans* and *M. incognita*. Furthermore, these effects were also observed in *C. elegans* brood size, growth, locomotion, and behavioral response.

## 2. Results

### 2.1. Molecular Characterization and Expression Analysis of the Nif3-family Protein YqfO03

**Figure 1:**
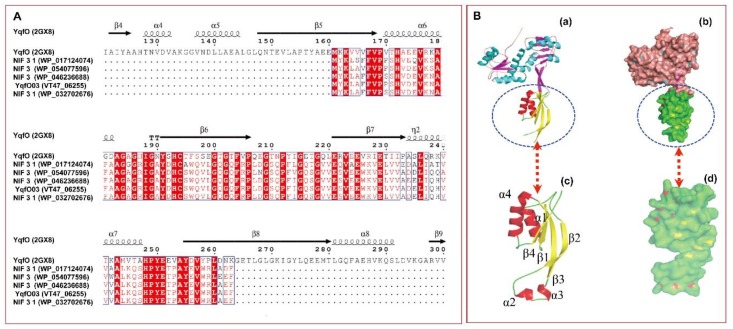
Sequence alignment of YqfO03 with several other Nif3-family proteins (**A**), and orthogonal and surface views of the ribbon and spatial structures of YqfO03 and *B. cereus* YqfO (**B**). In Figure 1A, sequence alignment with other Nif3 proteins, the additional Gen Bank accession numbers are given in parentheses; a tag number was used for YqfO03, and a PDB ID (2GX8) was used for YqfO. Secondary elements are shown above the alignment. Residues that are completely conserved are highlighted in solid red boxes. Those with similarity of >70% are labeled in red. The alignment was generated with the ClustalW2 program and used as the input for the ESPript program, Version 3.0 (http://espript.ibcp.fr/ESPript/cgi-bin/ESPript.cgi). In Figure 1B, homology model was built using 2GX8 as a 3D reference homolog model, (**a**)/(**b**): showing three-dimensional (3D) structure of YqfO; (**c**)/(**d**): YqfO03, showing similar parts both in orthogonal and surface views of the proteins. The colored α-helices (red) and β-sheet (yellow) constituting similar folds are shown in (**a**,**c**). The structural domains matching YqfO03 are indicated in the YqfO models by oval frames and arrows.

The software MP3 [29] was used to predict genes with virulence against *C. elegans* by a hybrid support vector machines (SVM) and hidden Markov model (HMM) approach. We selected a gene (annotated as “*VT47_06255*”) from the genome of the *P. syringae* wild-type strain MB03 (GenBank accession no. NZ_LAGV01000012.1), which had a high score for predicted virulence, as the target gene to investigate the pathogenic effect on *C. elegans* worms. The predicted VT47_06255 protein consists of 103 amino acid (AA) residues with a theoretical molecular mass of 11502.1 Da and a *pI* of 5.01. With regard to structural organization, this protein contains the distinguishing conserved domains of the Nif3-like protein YqfO from *Bacillus cereus* ATCC14579 [30] (Supplementary Figure S1A). No signal sequence was predicted at the N-terminus of this protein while using SignalP (http://www.cbs.dtu.dk/services/SignalP/). The predicted secondary structure of VT47_06255 shows multiple α/β folds (Supplementary Figure S1B), which coincide with the partial α/β motifs (i.e., partial β5, α6, β6, β7, α7, and partial β8) of the N-terminal domain of YqfO (Figure 1A). The AA sequence of VT47_06255 was aligned with the sequences of Nif3-family proteins from the genus *Pseudomonas*, such as proteins from *Pseudomonas gingeri* (GenBank accession no. WP_017124074), *Pseudomonas amygdali* (GenBank accession no. WP_054077596), *P. syringae* pv. *coryli* (GenBank accession no. WP_046236688), and *P. syringae* (GenBank accession no. WP_032702676), as well as YqfO (PDB ID: 2GX8) of *B. cereus* [30] (Figure 1A). Not surprisingly, VT47_06255 showed high similarity, of 90.29–100%, with proteins from the genus *Pseudomonas* and even higher similarity of 82.52% with the aligned region of YqfO (Figure 1A). Using a three-dimensional (3D) model of YqfO as a reference protein, the predicted tertiary structure of VT47_06255 protein was found to adopt the structure of YqfO well in both orthogonal and surface views of the proteins (Figure 1B), and several α/β folds between VT47_06255 protein [Figure 1B (a)] and YqfO [Figure 1B (b)] were seen to be apparently coordinated with the structural sites and configurations of the proteins. These results suggest that the VT47_06255 protein is a Nif3-family protein with high structural similarity to *B. cereus* YqfO; therefore, we named this protein YqfO03.

**Figure 2:**
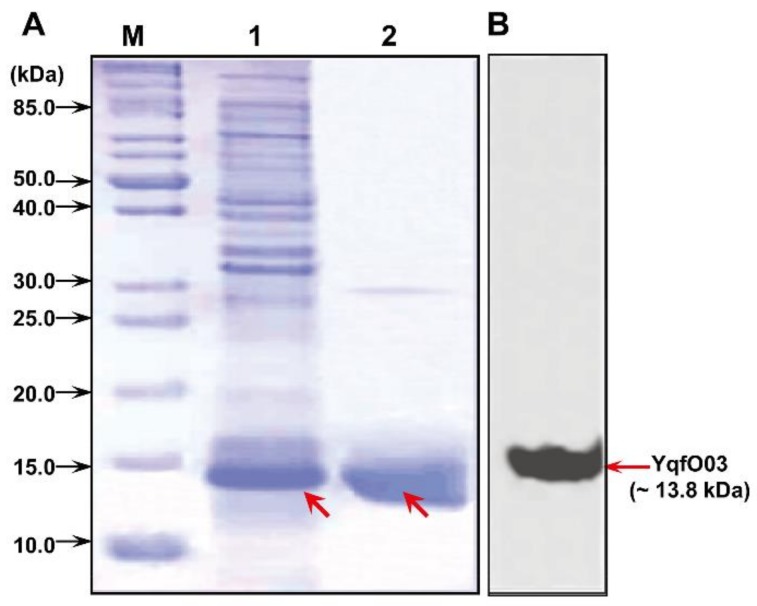
(**A**) SDS-PAGE and (**B**) western blot analyses of the purified YqfO03 protein. In (**A**), lane M: protein molecular weight marker; lane 1: whole-cell lysate of recombinant *E. coli* MB1171 cells upon IPTG induction; lane 2: purified YqfO03 protein.

The YqfO03-encoding gene (namely, *yqfO03*) was amplified by PCR and was used to construct the recombinant plasmid pMB-YqfO03 (Supplementary Figure S2). SDS-polyacrylamide gel electrophoresis (SDS-PAGE) analysis demonstrated that YqfO03 was expressed in recombinant *E. coli* cells as a fusion protein (with a His tag) with the predicted size (~13.8 kDa) (Figure 2A, lane 1, indicated by arrow). The expressed YqfO03 was purified and examined by SDS-PAGE gel (Figure 2A, lane 2, indicated by arrow) and western blot analyses (Figure 2B), which confirmed the size and purity of the purified protein.

### 2.2. Nematicidal Activity of Purified YqfO03 against C. elegans and M. incognita

**Figure 3:**
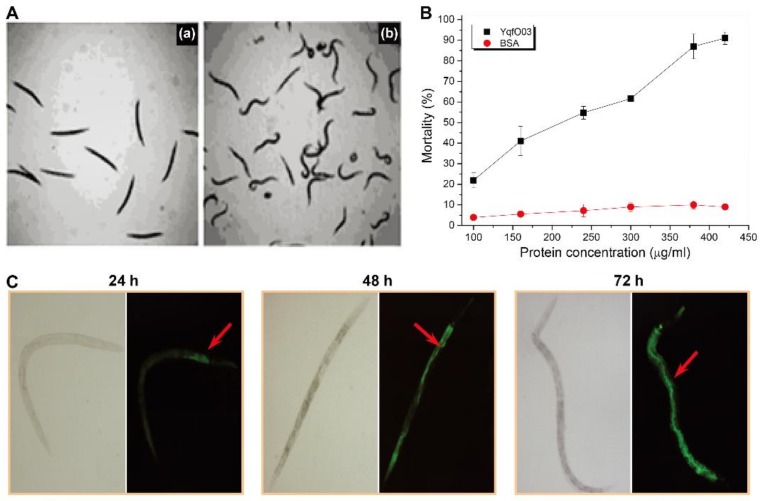
(**A**) Comparative morphology of *C. elegans* worms after treatment with YqfO03 protein, (**B**) nematicidal activity assay of purified YqfO03, and (**C**) in vivo distribution analysis of purified YqfO03 in worms. (**A**) (**a**) worms after feeding with 500 µg/mL YqfO03. Nematode death was confirmed by transferring the individuals into new plates containing sterile water, and the individual worm that did not respond after being touched several times with a platinum pick were marked as dead. The dead worms remained rigid and straight; (**b**) worms treated with bovine serum albumin (BSA). Worms were active and wavy with constant motion. (**B**) Mortality assays of *C. elegans* that were treated with various concentrations of purified YqfO03 at concentrations of 100, 160, 240, 300, 380, and 420 µg/mL, respectively. Error bars represent the standard deviations from the means of three independent experiments. (**C**) Worms were fed with purified FITC-labeled YqfO03 (200 µg/mL). Worm morphology and fluorescence signal were observed by fluorescence microscopy at 24, 48, and 72 h (dark background). FITC alone was used as the control (gray background).

We first evaluated the nematicidal activity of YqfO03 against free living nematode, *C. elegans*, using a lethality assay. *C. elegans* worms were killed with 100% mortality when treated with 500 μg/mL purified YqfO03 (Figure 3A). Therefore, we investigated the dose response of *C. elegans* mortality through a series of protein dilutions. As shown in Figure 3B, the mortality of the worms increased significantly with increasing protein concentrations from 100 μg/mL to 420 μg/mL. The calculated half-lethal concentration (LC_50_) of YqfO03 for *C. elegans* was 192.6 (160.40~222.90) µg/mL. Moreover, the determination of YqfO03 localization in *C. elegans* was performed by feeding *C. elegans* with either fluorescein isothiocyanate (FITC) labeled YqfO03 protein or FITC alone. We observed that the fluorescence signal of FITC-labeled YqfO03 was first emitted from around the mouth and esophagus and then from the first half of the nematode intestine, and finally the signal spread throughout the body (Figure 3C). Thus, these results indicate that the YqfO03 protein from *P. syringae* MB03 function as a nematicidal virulence factor and showed toxicity against *C. elegans*.

**Figure 4:**
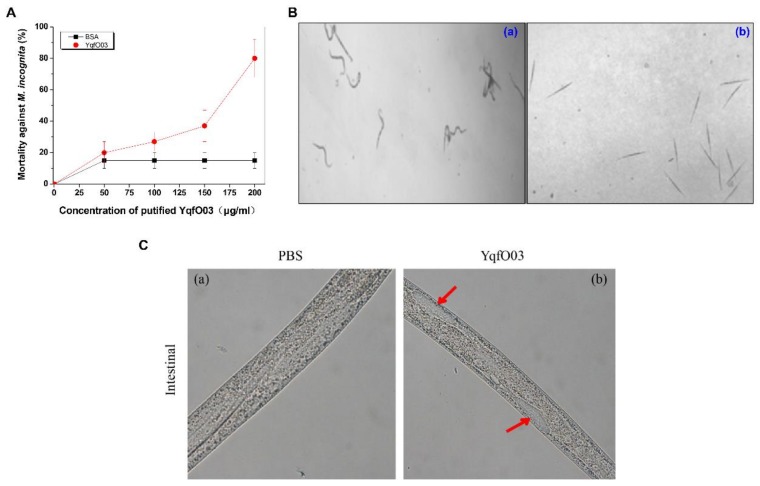
(**A**) Nematicidal activity assay of purified YqfO03, (**B**) Morphology of YqfO03-killed *M. incognita* worms, and (**C**) Micrographs of *M. incognita* after YqfO03 treatment. In (**A**), Mortality assays of *M. incognita* js2 that were incubated at different concentrations of 0, 50, 100, 150, and 200 µg/mL, of purified YqfO03 protein. BSA was used as the control. The mortality of the worms in each well was determined in 5 d. Error bars represent the standard deviations from the means of three independent experiments. In (**B**), (**a**) normal worms treated with BSA; (**b**) dead worms after incubated with YqfO03. In (**C**), (**a**) worms treated by phosphate-buffered saline (PBS) (pH 7.0) for 48 h; (**b**) worms treated by 500 µg/mL YqfO03 for 48 h. red arrows indicate a patch-like structure in the intestinal region of worm.

To determine the nematicidal lethality of YqfO03 against *M. incognita*, the purified YqfO03 at a final concentration of 0, 50, 100, 150, and 200 µg/mL was respectively incubated with *M. incognita* worms to observe its lethal activity. As shown in Figure 4A, following the increasing concentration of YqfO03, the mortality of *M. incognita* second-stage juveniles (J_2_) worms increased. The calculated LC_50_ of YqfO03 was 175.4 µg/mL. After treatment with 200 µg/mL YqfO03, all *M. incognita* worms were found dead with rigid and straight bodies, whereas when treated with bovine serum albumin (BSA), the worms were vigorous and still in motion (Figure 4B). The intestinal morphology was examined using a light microscope. As shown in Figure 4C, the intestinal tract of worms that were treated with YqfO03 appeared to be pathologically changed, as some patch-like special structures were clearly visible (Figure 4C(b)), which might be due to the destruction of intestinal tract of worms by YqfO03. Thus, the YqfO03 protein also showed significant toxicity against *M. incognita*.

### 2.3. Effects of Purified YqfO03 on Brood Size and Growth of C. elegans

**Figure 5:**
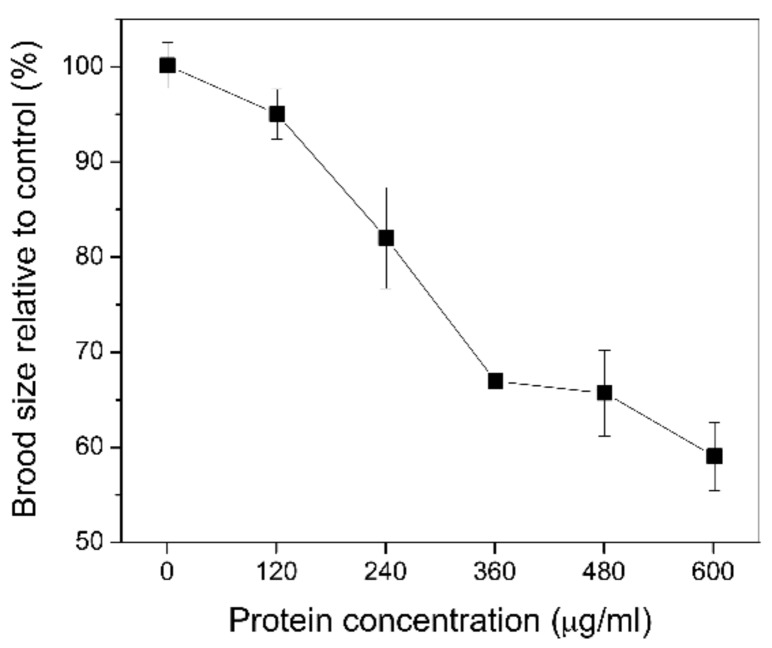
Brood size assay of *C. elegans* using different concentrations of the purified YqfO03. The assay was measured in 3 d. Error bars represent the standard deviations from the mean of three independent experiments.

**Figure 6:**
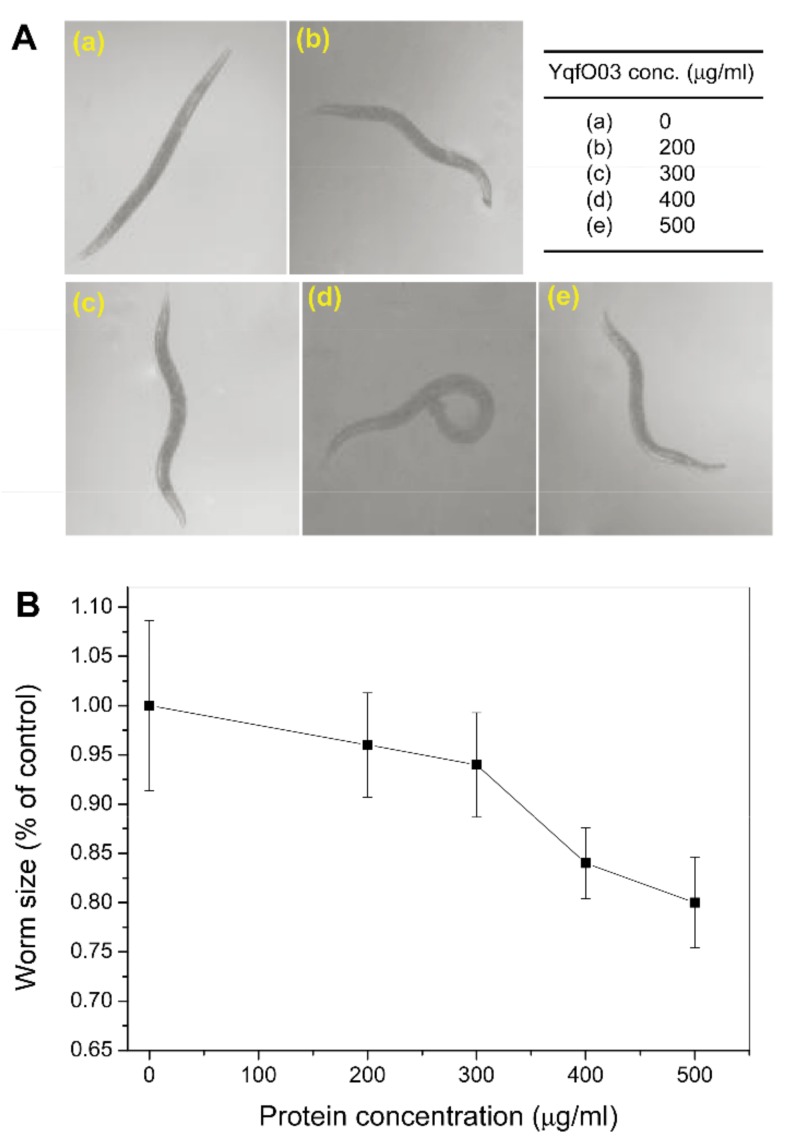
Growth assay of L_1_ larvae of *C. elegans* with YqfO03 protein treatment. (**A**) Wild-type L_1_ worms were cultured in gradient doses of the YqfO03 protein in a concentration range of 0–500 µg/mL, were incubated at 20 °C for 60 h, and then were photographed using a light microscope. (**B**) The size of worms cultured in 0–500 µg/mL of the YqfO03 protein as a percentage of the size of worms cultured in the absence of the YqfO03 protein. The average size of worm was calculated using the software NIH Image J 1.3. Data represent the averages of 20 measurements at each protein concentration. Error bars represent standard deviations.

To assess the effect of the Nif3-related YqfO03 purified protein on brood size and growth of *C. elegans*, different concentrations of the purified YqfO03 protein were incubated with the worms for 3 d. Figure 5 shows that YqfO03 had significant impact on the brood size of *C. elegans*; increasing concentrations of YqfO03 caused a coordinated decline of brood size. For the growth assay, the synchronized first-stage (L_1_) larvae were fed with serial dilutions of the purified YqfO03 protein. As shown in Figure 6A, while low concentrations of YqfO03 (200–300 µg/mL) had a limited suppressive effect on worm size, increasing the concentration of YqfO03 to 400–500 µg/mL caused the visible suppression of worm growth, with an approximately 20% decrease in growth size at 500 µg/mL YqfO03, as determined by worm-size measurement (Figure 6B).

### 2.4. Effect of YqfO03 on the Motility of C. elegans

**Figure 7:**
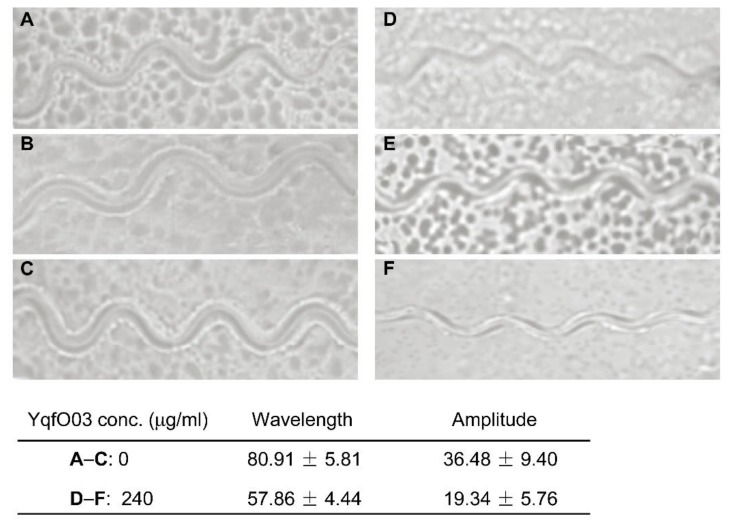
Effect of purified YqfO03 on *C. elegans* motility. In (**A**–**C**), track patterns of worms on S medium in the absence of YqfO03 (negative control). In (**D**–**F**), track patterns of worms on S medium containing 240 µg/mL of purified YqfO03 protein.

To analyze the effects of YqfO03 on *C. elegans* motility, the worms were placed on a lawn of *E. coli* OP50 cells and the movement of the worms was measured. There was a clear difference between untreated worms (Figure 7A–C) and the worms that were treated with 240 µg/mL purified YqfO03 (Figure 7D–F). The effect of YqfO03 on worm motility was analyzed by recording the amplitude and wavelength values of worm movement. The measured values showed that both the wavelength and amplitude of the body waves of the worms were significantly reduced as compared with those of the control (Figure 6). These results suggest that YqfO03 caused prominent defects in *C. elegans* motility as the worms moved through the *E. coli* lawn.

### 2.5. Effect of YqfO03 on the Behavioral Responses of C. elegans

**Figure 8:**
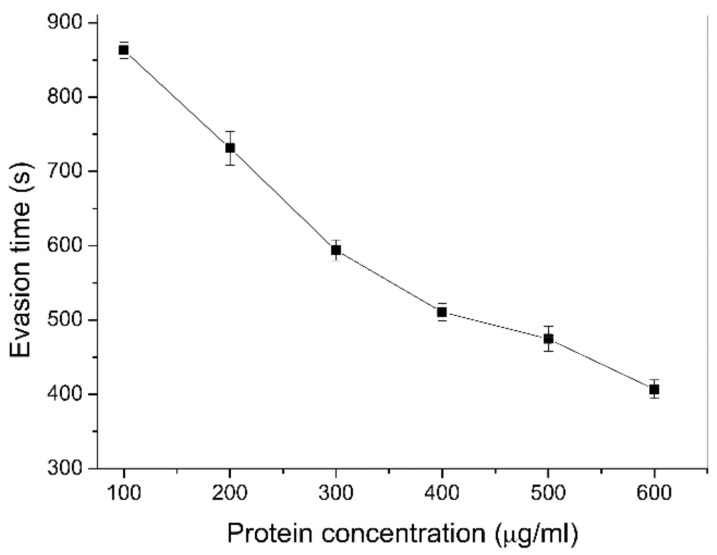
Physical evasion time of L_4_ worms after exposure to different doses of the YqfO03 protein. Error bars represent the standard deviations of three evasion assays.

The behavioral response of *C. elegans* to different concentrations of purified YqfO03 was examined by measuring the evasion time of the worms. The evasion time was the time needed by an individual worm to leave a small “spot” in the presence or absence of a protein. As shown in Figure 8, as the protein concentration increased, *C. elegans* fled the lawn at a higher rate than the control. Therefore, exposure to YqfO03 led to a faster evasion response of *C. elegans* than exposure to the negative control.

## 3. Discussion

Many virulence factors have been reported to have nematicidal activities, particularly from *Bacillus* spp. The Cry proteins and other proteases are the most characterized bacterial proteins with potent nematicidal activities against *C. elegans* and *M. incognita* [10,11,12]. Since resistance to these factors can easily occur, there is a need to find new virulence factors that have significant nematicidal activity against PPNs.

A variety of proteins from *P. aeruginosa* are involved in the pathogenesis of the nematode *C. elegans* [20,21,22], however, little is known about the nematicidal activity of *P. syringae* against this nematode. Likewise, limited information is available regarding the nematicidal proteins that are capable of killing PPN, *M. incognita*. In the current study, we attempted to explore a new nematicidal factor in the *P. syringae* strain MB03. The significant nematicidal activity of a Nif3-family protein “YqfO03” isolated from *P. syringae* strain MB03, was investigated against *C. elegans* and important PPN *M. incognita.* Furthermore, we determined the diverse nematicidal activities of this protein against *C. elegans*, which include effects on brood size, growth, motility, and behavioral response of *C. elegans*, suggesting the potential use of this protein as new bio-agent to control PPN. To the best of our knowledge, this study is the first report of nematicidal activity of a protein from the Nif3-family.

Nif3-family proteins are highlighted as a group of hypothetical proteins with unknown function, To date, the crystal structures of several bacterial Nif3 proteins have been determined, including the structures of YqfO from *B. cereus* (PDB id: 2GX8) [30], YbgI from *E. coli* (PDB id: 1NMP) [31], SA1388 from *Staphylococcus aureus* (PDB id: 2NYD) [32], and TTHA1606 from *Thermus thermophilus* HB8 (PDB id: 2YYB) [33]. Thus, the structural prediction of YqfO03 by comparing with available Nif3 protein structures could help us to understand the mechanism of nematicidal activity of this protein. The crystal structure of YqfO contains three molecules per asymmetric unit. Unlike the two-domain architecture of YbgI, the YqfO protein contains three structural domains (D1, D2, and D3). D1 and D3 are similar to the α/β/α sandwich topology as compared to YbgI, with a central, mixed β-sheet bounded by two helices on the top and two helices on the bottom, forming a hexameric ring, which is a substrate-binding site, as it is certainly large enough to accommodate a nucleotide or small peptide [30]. The other more likely substrate-binding site is found on the opposite side of the dimetal-binding pocket, which could be a co-catalytic site having two proximate metal ions (including zinc ions) and this structure is similar to other proteins with proximate zinc ions, such as phosphoesterases, aminopeptidases, and β-lactamases [34]. Therefore, the YqfO protein is suggested to be a metalloenzyme whose activity may be regulated by allosteric ligands [30]. Interestingly, these phosphoesterases, amino peptidases are also well characterized in *P. aeruginosa* that can cause extensive tissue damage, invasion, and interfere with host proteins and can degrade numerous host defense proteins [35]. Moreover, the Nif3-related protein was found to exhibit an inhibitory effect on the pathogen *Fusarium oxysporum* in addition to other degrading enzymes from *B. subtilis* for the control of *Fusarium oxysporum* [28]. The YqfO03 protein from *P. syringae* MB03 showed high homology to YqfO from *B. cereus (*Figure 2). Therefore on these structural based functional properties of YqfO, we can speculate that YqfO03 is also a kind of degrading enzyme that is responsible for lethality against *C. elegans* and *M. incognita* (Figure 3 and Figure 4) and cause damage to the intestinal tract of worms (Figure 4C). Thus, YqfO03 could serve as a degrading enzyme that is involved in degrading the intestinal tracts and/or might degrade the host defense proteins which ultimately leads to worm’s death. Further studies are required to elucidate the biochemical properties and molecular action mechanisms of YqfO03 targeting the host nematode.

Additionally, we evaluated various nematicidal properties of YqfO03 against *C. elegans*. Our results indicated that the YqfO03 protein can suppress brood size, and, to a lesser extent, can inhibit the growth of *C. elegans* worms as well. Moreover, the presence of YqfO03 led to abnormalities in *C. elegans* motility. Previous studies with the Cry6Aa2 showed that this toxin also had adverse effects on nematodes motility [36]. Generally, *C. elegans* tracks on *E. coli* plates in a perfect sinusoidal path. The apparent defects in the amplitude and wavelength of the sinusoidal tracks of *C. elegans* after treatment with YqfO03 suggest that this protein may intoxicate the nematodes and adversely affect the health of the nematodes. Since growth, brood, and locomotion are key steps for its survival and reproduction in nematode life cycle, so the suppression of any developmental step of nematodes could lead to the interruption of their intact life cycle, and effectively control their reproduction and damages in the hosts. Since PPNs can absorb various molecules directly from the environment, including peptides and proteins, [6], the YqfO03 protein can easily be taken up by *M. incognita* due to its smaller size. These properties are significant for the bio-control of *M. incognita* worms that are otherwise difficult to feed with a large size of toxins [4].

When encountering the pathogens, nematodes can either move away from these potentially toxic pathogens or can reduce their ingestion rates. We used different doses of purified YqfO03 on *E. coli* lawn plates and found that the *C. elegans* escape the lawn containing YqfO03 faster than the control plates. Similar results for *C. elegans* were also observed in response to treatment with some *Pseudomonas* spp. and *Bacillus* toxins [25,36]. Therefore, pre-plant treatment with YqfO03 could lower the populations of nematode to prevent major nematode damage, especially during the early developmental stages of plant. The present study confirms that YqfO03 from *P. syringae* MB03 is a nematicidal virulence factor against *C. elegans* and *M. incognita*. Nematicidal activity is probably attributed to many factors, including proteins, toxic peptides, or other metabolic products. The comparative genomic analyses indeed revealed multiple potential nematicidal virulence factors in *P. syringae* MB03 in addition to YqfO03 [18]. We have also determined the role of Pyoverdine in MB03, which showed the efficient killing of *C. elegans* (data is not published) and other nematicidal factors, such as RTx and Spr proteins, are attributable to the virulence of MB03 against nematodes. Therefore, this and other putative virulence factors need further research to better understand the molecular mechanism of MB03 against nematode. The protection of plants by the direct application of this YqfO03, or constructing *Bacillus subtilis* or other commercial strains transformed with the *yqfO03* gene for RKN control; additionally, the *yqfO03* gene could be developed as transgenic plants with increased nematicidal activity for the efficient control of RKN.

## 4. Materials and Methods

### 4.1. Bacterial Strains, Plasmids, and Culture Conditions

The bacterial strains and plasmids that were used in this study are listed in Supplementary Table S1. The *P. syringae* subsp. *syringae* wild-type strain MB03 was isolated and identified in a previous study [37]. The *P. syringae* cultures were grown on nutrient agar medium containing 1% peptone, 0.5% yeast extract, 1% NaCl, and 1.5% agar. *E. coli* strains were routinely grown at 37 °C in lysogeny broth (LB) medium [38], while recombinant *E. coli* cells were grown in LB medium containing 100 µg/mL (final concentration) ampicillin (Amp). The *C. elegans* N_2_ wild-type strain was provided by the *Caenorhabditis* Genetics Center (CGC) (College of Biological Sciences, University of Minnesota, MN55108, USA) and it was maintained at 20 °C on nematode growth medium (NGM) agar plates with *E. coli* OP50 as food [39]

The root knot nematode *M. incognita* was propagated by infecting the tomato plants (Lycopersicon esculentum Mill cv. 144) in the greenhouse. Sixty days (approx.) later, tomato roots were harvested. *M. incognita* egg masses (galls) were handpicked under a dissecting microscope from tomato roots and then sterilized with bleach. To set up bioassays of *M. incognita* mortality, eggs were recovered from tomato plants by shaking *M. incognita*-infected roots in a 1:9 dilution of bleach solution for 3 min in a flask. Eggs were collected on a 25-μm mesh and were then further bleached twice for 10 min with a 1:5.7 dilution of bleach supplemented with 0.02% Tween-20. The eggs were then washed three times with sterile double-distilled water, the eggs were harvested by centrifugation at 500× *g* for 1 min. Eggs were hatched at room temperature for two days in 100 mg/L carbenicillin, and second-stage juvenile (J_2_) worms were collected. These J_2_ worms were used for *M. incognita* mortality assay.

### 4.2. Gene Cloning, Protein Expression, and Purification

The genomic DNA of *P. syringae* MB03 was extracted using the Genomic DNA Purification Kit (Promega) and was used as a template for polymerase chain reaction (PCR) of the gene *yqfO03*. The PCR primers were 5′–TCACTGCAGCTGTGTACAAGCTCGCCTTC–3′ (*Pst*I site underlined) and 3′–CCGGAATTCTCAGAACTCTGCCAGCC–5′ (*Eco*RI site underlined). The amplified 332-bp *yqfO03* gene was digested with *Pst*I and *Eco*RI and it was ligated at the same sites in the *E. coli* expression vector pTrcHis B (Invitrogen), yielding the recombinant plasmid pMB-YqfO03 (4704 bp, Supplementary Figure S1). This plasmid was used to transform *E. coli* JM109 competent cells according to standard procedures [38]. The recombinant *E. coli* cells were inoculated into LB medium containing ampicillin (100 µg/mL) and the cells were grown with shaking at 37 °C for 1.5 h. When the culture reached an OD_600_ of 0.2, isopropyl-β-d-thiogalactoside (IPTG) was added to the culture at a final concentration of 0.3 mmol/L to induce protein expression. The induced cultures were grown for an additional 8 h at 30 °C. These cells were collected by centrifugation, resuspended in phosphate-buffered saline (PBS) (0.8% NaCl, 0.02% KCl, 0.14% Na_2_HPO_4_, 0.03% KH_2_PO_4_, pH 7.0), and homogenized (NS100IL 2K, Niro Soavi, Germany). The target protein was purified using a His-Bind column (31314; Qiagen, Hilden, Germany), according to the manufacturer’s instructions. The molecular weight of the purified protein was analyzed by 15% SDS-PAGE, and the protein concentration was measured by the Bradford method using BSA as a standard [40].

### 4.3. Western Blot Analysis

Western blot analyses were conducted using an anti-His-tag primary antibody and an AB clonal anti-mouse IgG (H + L) horseradish peroxidase (HRP)-conjugated secondary antibody that was purchased as a DAB kit (Ling Fei Biological Limited, Wuhan, China). The protein samples were separated on a 15% SDS-PAGE gel and then transferred to a nitrocellulose membrane. The membrane was incubated overnight with a 1:11,000 dilution of the anti-His antibody. After washing, the membranes were incubated with the HRP-conjugated secondary antibody at a 1:1500 dilution. The signal was visualized with an enhanced chemiluminescence substrate (Bio-Rad, Hercules, CA, USA).

### 4.4. C. elegans Mortality Assay

*C. elegans* mortality assay was performed using vigorous synchronized L4-stage *C. elegans* worms following the method that was described by Bischof et al. [39]. Briefly, 5 µL of culture containing 30 to 50 worms was added to each well of a 96-well microtiter plate. Each well contained 140 µL of S medium with *E. coli* OP50, as a food source 5 µL of 8 mM FUdR (5-fluorodeoxyuridine), 0.6 µL of ampicillin, and the desired amount of the purified YqfO03 protein or BSA as a control. Different concentrations of YqfO03 protein were used to observe a dose-dependent effect. The 96-well plate was placed in an enclosed box and wrapped with parafilm to provide a humid environment. Subsequently, the mortality in each well was observed and counted under an inverted microscope (Olympus IX73, Tokio, Japan) after incubation at 20 °C for five days. Motile worms were marked as alive, while immotile worms were transferred to sterile water and the worms that did not respond after being touched several times with a platinum pick were marked as dead. Data were plotted as the percentage of dead worms versus the concentration of Nif3-related YqfO03. The LC_50_ values were determined by PROBIT analysis using SPSS, and the bioassay were repeated a minimum of three times.

### 4.5. Mortality Assay of M. incognita

The mortality assay of *M. incognita* J_2_ was performed in a 96-well microtiter plate following method with little modification, as described previously [39]. A single well containing 30 to 40 vigorous *M. incognita* J_2_ worms, incubated with purified YqfO03 protein, and added PBS to a total volume of 200 µL. The 96-well culture plates were placed in a box to maintain humidity. The whole assay was incubated at 20 °C for 5 d. Whether *M. incognita* J_2_ worms were alive was also determined based on the movement of worms. A visibly moving J_2_ was marked as alive. The nonmoving J_2_ worms were transferred to sterile water and they were gently touched with a platinum pick to observe the response. If the worm failed to respond after three touches, it was deemed dead. The LC_50_ values were determined by PROBIT analysis using SPSS, and the bioassay were repeated a minimum of three times.

### 4.6. Brood Size Assay and L_1_ Growth Assay

The brood size assay was performed in 96-well microtiter plates. Each well was coated with 5 µL of an *E. coli* OP50 culture in S medium at an OD_600_ of 2.0 and it contained a single L_4_ worm. The purified YqfO03 protein at different concentrations was then added to the wells. Next, sufficient S medium was added to each well to bring the total volume to 120 µL [41]. Five wells were assayed for each protein concentration. Microtiter plates were placed at 25 °C for 3 d. Then, the total numbers of eggs in each condition were determined. Assays were repeated a minimum of three times.

The growth assays for the L_1_ *C. elegans* were performed by following a previously described method [39]. Briefly, 10 µL of *E. coli* OP50 at an OD_600_ of 2.0, used the desired amount of purified YqfO03 protein at five different concentrations, 0.1 µL of chloramphenicol, and 5 µL of 20–30 L_1_ stage worms were added to each well of a 96-well plate. For the negative control, S medium was used instead of the protein solution. Three replicate experiments were performed for each protein concentration. The 96-well plates were placed at 20 °C for 60 h. After incubation, the worms were gently mixed; 3–5 µL was pipetted onto a 2% Agarose pad containing 15 mmol/L sodium azide as an anesthetic; and, a coverslip was placed on top. At least 20 worms for each concentration were photographed under a 100× magnification on compound microscope, and the lengths of these worms were calculated using NIH Image J1.33 software. Finally, the average length of the worms was plotted against the average length of the control group for comparison.

### 4.7. Effect of Purified Protein on Nematode Motility

*C. elegans* motility assays were performed according to a previously described method [42] with some modifications. Individual L_4_ worms were transferred into wells of a 96-well microtiter plate containing 20 µL of *E. coli* OP50 resuspended in S medium at an OD_600_ of 3.0, 0.1 µL of chloramphenicol, 2.5 µL of 8 mM FUdR, and different concentrations of purified YqfO03 were used, added the required amount of protein in a total of 100 µL of each well. In the control group, the protein solution was replaced with S medium. Afterward, the plates were incubated for 24 h at 20 °C. After incubation, the worms were transferred to fresh plates with *E. coli* OP50 lawns and allowed to cut tracks for 10–20 min before the paths were measured. The control nematodes and nematodes treated with the various protein concentrations were photographed, and the amplitude and wavelength of the nematode tract were calculated.

### 4.8. Behavioral Response of C. elegans

The assay was performed according to a previously described protocol [43]. Overnight cultures of *E. coli* OP50 (10 µL) were used to coat the wells of 24-well plates containing 3.4% agar. The wells were dried at 20 °C overnight. Five different purified protein concentrations were placed in the middle of the *E. coli* lawn. Three replicate experiments were performed for each protein dilution. S medium was placed in middle of the *E. coli* lawn for the negative control. Next, the samples were left to dry for 2 h. Then, an individual nematode was placed in the middle of each lawn with a worm picker. Subsequently, the time that was needed for the entire body of each worm to leave the spot containing either the protein or the S medium was recorded. If the worm did not leave the spot within 10 min, observation was stopped, and 10 min was noted as the time taken by this individual.

### 4.9. Microscopy

Images of worms and tracks in plates were captured with an Olympus digital camera on an Olympus dissection microscope. A fluorescence microscope (Olympus IX83, Tokyo, Japan) was used to observe the fluorescence of FITC-labeled protein after feeding the protein to the worms.

### 4.10. Bioinformatics

Sequence similarity and conserved domain homology were determined using NCBI database and the ExPASy program. The 3D homology model was built using PyMOL and the secondary structure was described using the ESPript program, Version 3.0 (http://espript.ibcp.fr/ESPript/cgi-bin/ESPript.cgi).

### 4.11. Data Analysis

Data analysis was performed using SPSS (Statistical Package for the Social Sciences) software, Version 13.0. LC_50_ values were calculated using PROBIT analysis 48 and they are shown as the mean ± SD (*n* = 3). Graphs were prepared using Origin 8 software (Origin Lab Corp., Northampton, MA, USA).

## 5. Conclusions

In conclusion, the data we present here shows that the Nif3-family protein YqfO03 from *P. syringae* MB03 has nematicidal activity against *C. elegans* and *M. incognita.* Furthermore, Purified YqfO03 protein causes reduced brood size, inhibits L1 growth, and causes abnormal locomotion of *C. elegans*. Additionally, *C. elegans* showed strong physical evasion behavior toward this protein. Our findings suggest that the Nif3-family protein YqfO03 could be used as a novel alternative sustainable agent for PPN control.

## Supplementary Materials

Supplementary Materials can be found at http://www.mdpi.com/1422-0067/19/12/3915/s1.
