# Supplementary material for: The Nif3-Family Protein YqfO03 from Pseudomonas syringae MB03 Has Multiple Nematicidal Activities against Caenorhabditis elegans and Meloidogyne incognita
Source: Int J Mol Sci. 2018 Dec 6;19(12):3915. doi: 10.3390/ijms19123915 (PMC6321441; doi:10.3390/ijms19123915)
Supplement: Supplementary file 1 [file ijms-19-03915-s001.zip › Supplementary Material.pdf]

Supplementary Materials

The Nif3-family protein YqfO03 from *Pseudomonas syringae* MB03 has diverse anti-nematodal activity against *Caenorhabditis elegans*

Abdul Manan [1,2], Zahoor Ahmad Bazai [3], Jin Fan [1], Huafu Yu [1] and Lin Li [1,*]

[1] State Key Laboratory of Agricultural Microbiology, College of Life Science and Technology, Huazhong Agricultural University, Wuhan 430070, China; manan_haqyar@yahoo.com (A.M.); 38748853@qq.com (J.F.); yuhuafu20093919@163.com (H.Y.); lilin@mail.hzau.edu.cn (L.L)

[2] Center for Advance Studies Vaccinology and Biotechnology, University of Baluchistan, Quetta, Pakistan

[3] Department of Botany University of Baluchistan, Quetta, Pakistan; z_Bazai@yahoo.com

* Correspondence: lilin@mail.hzau.edu.cn; Tel.: +86-27-87286952; Fax: +86-27-87280670

Table S1. Bacterial strains and plasmids used in this study

Strains and plasmids	Phenotypes [a]	Source
E. coli JM109	*recA*1 e*ndA*1 *gyrA96 thi hsdR17 supE44 relA1 Δ(lac-proAB)/F' [traD36 proAB+ lacIq lacZΔM15]*	Invitrogen
E. coli Top10	*recA1 endA1 gyrA96 thi hsdR17 supE44 relA1 Δ(lac-proAB)/F' [traD36 proAB+ lacIq lacZΔM15]*	Invitrogen
E. coli OP50	The food source used for C. *elegans* N2	CGC
E. coli MB-YqfO03	An *E. coli* TOP10 construct harboring pMB-YqfO03	This study
P. syringae MB03	Wild-type strain with highly ice-nucleating activity	Li *et al*., 2012
pTrcHis B	Amp^r, *E. coli* expression vector, 4412 bp	Invitrogen
pMB-YqfO03	Amp^r, the recombinant pTrcHis B carrying the gene *yqfO03*, 4704 bp	This study

Note: [a] Amp^r, ampicillin resistance; *yqfO03*, a Nif3-like protein gene from *P. syringae* MB03; CGC, the *Caenorhabditis* Genetics Center, College of Biological Sciences, University of Minnesota, MN55108, USA.

References:

[1] Li, Q.; Yan, Q.; Chen, J.; He, Y.; Wang, J.; Zhang, H.; Yu, Z.; Li, L. Molecular characterization of an ice nucleation protein variant (InaQ) from *Pseudomonas syringae* and the analysis of its transmembrane transport activity in *Escherichia coli*. *Int. J. Biol. Sci.* **2012**, *8*, 1097−1108.

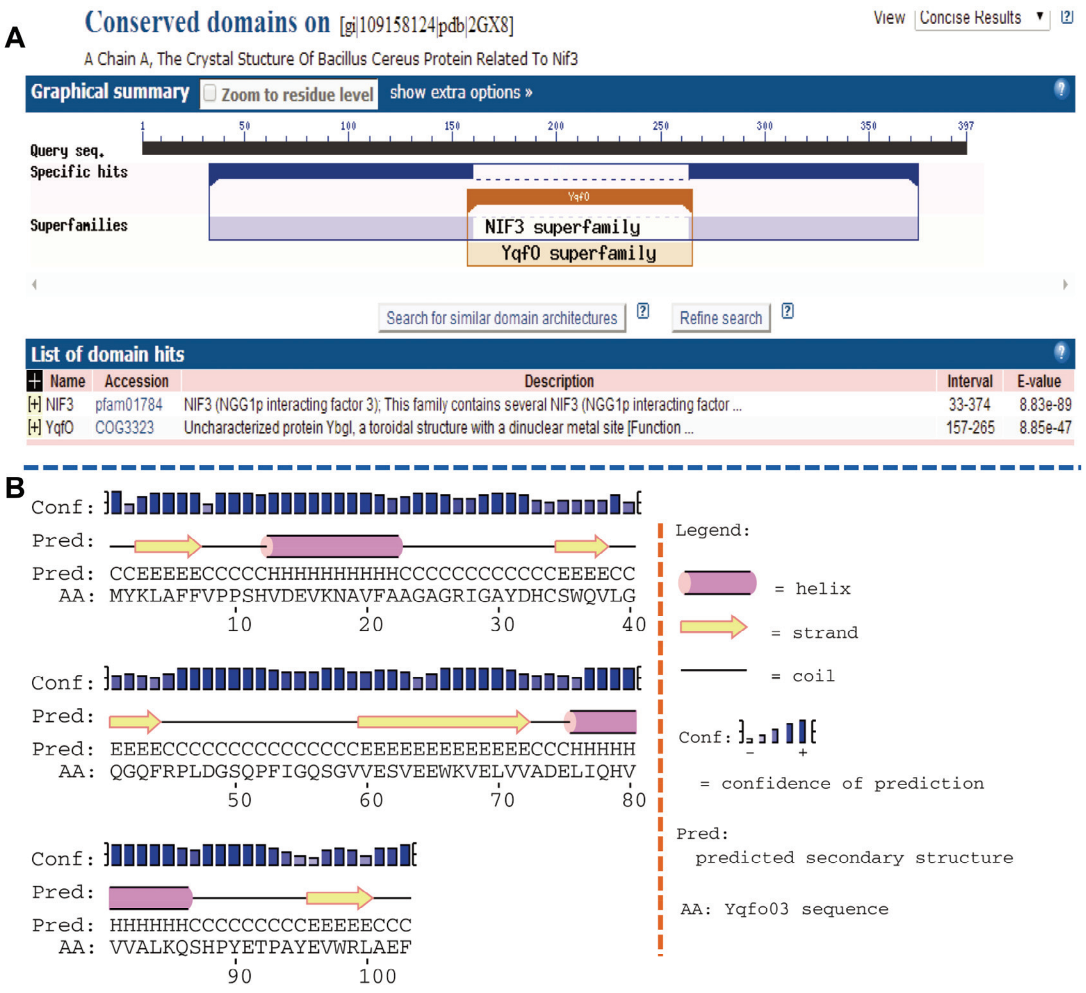

Figure S1. Comparison of the conserved domains of YqfO03 with the Nif3 protein YqfO from *Bacillus cereus* (A) and the predicted secondary structure of the YqfO03 protein (B).

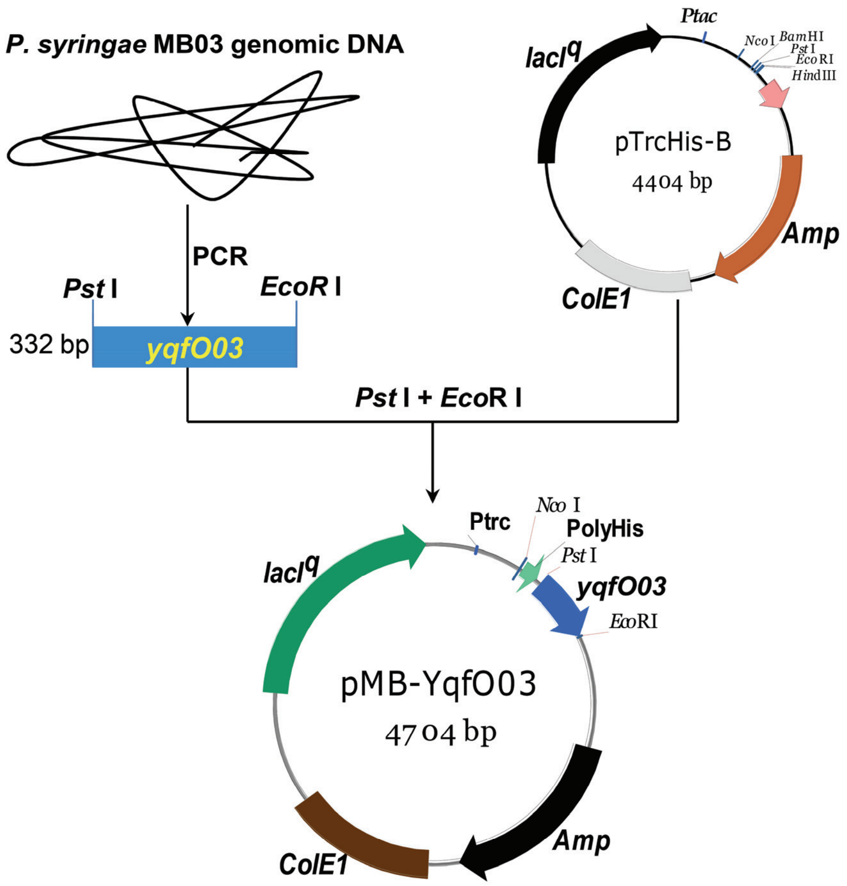

Figure S2. Schematic illustration of the construction of the recombinant plasmid pMB-YqfO03 expressing the nematicidal gene *yqfO03*.
